# Plasma Acylcarnitines during Pregnancy and Neonatal Anthropometry: A Longitudinal Study in a Multiracial Cohort

**DOI:** 10.3390/metabo11120885

**Published:** 2021-12-17

**Authors:** Yiqing Song, Chen Lyu, Ming Li, Mohammad L. Rahman, Zhen Chen, Yeyi Zhu, Stefanie N. Hinkle, Liwei Chen, Susanna D. Mitro, Ling-Jun Li, Natalie L. Weir, Michael Y. Tsai, Cuilin Zhang

**Affiliations:** 1 Department of Epidemiology, Indiana University Richard M. Fairbanks School of Public Health, Indianapolis, IN 46202, USA; yiqsong@iu.edu; 2 Department of Population Health, Division of Biostatistics, NYU Grossman School of Medicine, NYU Langone Health, New York, NY 10016, USA; Chen.Lyu@nyulangone.org; 3 Department of Epidemiology and Biostatistics, School of Public Health, Indiana University, Bloomington, IN 47405, USA; li498@indiana.edu; 4 Department of Population Medicine and Harvard Pilgrim Healthcare Institute, Harvard Medical School, Boston, MA 02215, USA; mlr782@mail.harvard.edu; 5 Division of Population Health Research, *Eunice Kennedy Shriver* National Institute of Child Health and Human Development, National Institute of Health, Bethesda, MD 20817, USA; chenzhe@mail.nih.gov (Z.C.); susanna.mitro@nih.gov (S.D.M.); 6 Kaiser Permanente Northern California Division of Research, Oakland, CA 94612, USA; Yeyi.Zhu@kp.org; 7 Department of Biostatistics, Epidemiology and Informatics, Perelman School of Medicine, University of Pennsylvania, Philadelphia, PA 19104, USA; stefanie.hinkle@pennmedicine.upenn.edu; 8 Department of Epidemiology, Fielding School of Public Health, University of California, Los Angeles, CA 90095, USA; cliwei86@g.ucla.edu; 9 Department of O&G, Yong Loo Lin School of Medicine, National University of Singapore, Singapore 119228, Singapore; obgllj@nus.edu.sg; 10 Department of Laboratory Medicine and Pathology, University of Minnesota, Minneapolis, MN 55455, USA; weirx065@umn.edu (N.L.W.); tsaix001@umn.edu (M.Y.T.)

**Keywords:** acylcarnitine, birthweight, body length, sum of skinfolds, sum of body circumference, pregnancy, women, gestational weeks, neonatal anthropometry

## Abstract

As surrogate readouts reflecting mitochondrial dysfunction, elevated levels of plasma acylcarnitines have been associated with cardiometabolic disorders, such as obesity, gestational diabetes, and type 2 diabetes. This study aimed to examine prospective associations of acylcarnitine profiles across gestation with neonatal anthropometry, including birthweight, birthweight z score, body length, sum of skinfolds, and sum of body circumferences. We quantified 28 acylcarnitines using electrospray ionization tandem mass spectrometry in plasma collected at gestational weeks 10–14, 15–26, 23–31, and 33–39 among 321 pregnant women from the National Institute of Child Health and Human Development (NICHD) Fetal Growth Studies-Singletons. A latent-class trajectory approach was applied to identify trajectories of acylcarnitines across gestation. We examined the associations of individual acylcarnitines and distinct trajectory groups with neonatal anthropometry using weighted generalized linear models adjusting for maternal age, race/ethnicity, education, parity, gestational age at blood collection, and pre-pregnancy body mass index (BMI). We identified three distinct trajectory groups in C2, C3, and C4 and two trajectory groups in C5, C10, C5–DC, C8:1, C10:1, and C12, respectively. Women with nonlinear decreasing C12 levels across gestation (5.7%) had offspring with significantly lower birthweight (−475 g; 95% CI, −942, −6.79), birthweight z score (−0.39, −0.71, −0.06), and birth length (−1.38 cm, −2.49, −0.27) than those with persistently stable C12 levels (94.3%) (all nominal *p* value < 0.05). Women with consistently higher levels of C10 (6.1%) had offspring with thicker sum of skinfolds (4.91 mm, 0.85, 8.98) than did women with lower levels (93.9%) during pregnancy, whereas women with lower C10:1 levels (12.6%) had offspring with thicker sum of skinfolds (3.23 mm, 0.19, 6.27) than did women with abruptly increasing levels (87.4%) (*p* < 0.05). In conclusion, this study suggests that distinctive trajectories of C10, C10:1, and C12 acylcarnitine levels throughout pregnancy were significantly associated with neonatal anthropometry.

## 1. Introduction

Pregnant women undergo systemic and dynamic physiological adaptations to meet the nutritional and metabolic demands of both the mother and developing fetus [1]. Emerging evidence suggests that maintenance of healthy mitochondrial function is essential to ensure adequate fetal nutrient and energy supply for growth and development, since mitochondria utilize a variety of substrates, including fatty acids, carbohydrates, ketones, and certain amino acids [2,3,4]. Alterations in mitochondrial energy metabolism, thus, may affect perinatal health outcomes.

Acylcarnitines—also known as fatty acid-carnitine esters—are a class of lipids, most of which (C2–C26) are derived through fatty acid β oxidation (FAO), an important biochemical process that generate energy from fat [5,6,7,8]. Acylcarnitines are detectable in plasma, and their levels vary as a function of various metabolic pathways related to biosynthesis, turnover, and transport, as well as renal handling [6,9,10]. Elevated circulatory acylcarnitines have been identified as surrogates of mitochondrial dysfunction, reflective of inefficient coordination across FAO, the tricarboxylic acid cycle, and the electron transport chain in both animal models and human studies [5,8,11]. Acylcarnitine profiling analysis using plasma or other biospecimens, especially long-chain acylcarnitines, has a clinical application in neonatal screening to identify inborn genetic defects of mitochondrial FAO and electron transport chain [9,10]. Elevated plasma levels of various acylcarnitines were also associated with cardiometabolic disorders, such as obesity, impaired glucose tolerance, insulin resistance, and type 2 diabetes [5,7,11,12,13]. A few studies on pregnant women reported that elevated acylcarnitines were associated with gestational diabetes mellitus (GDM) and childhood obesity [14,15,16,17,18]. However, these studies had small sample sizes [14,17] and/or lacked serial measurements of acylcarnitines [15,16,17,18]. In particular, there is a lack of longitudinal data on time-specific acylcarnitine profiling across pregnancy in association with neonatal anthropometric measures, which is critical as maternal metabolism changes greatly across gestation.

We hypothesized that different levels or distinct patterns in plasma acylcarnitines, during critical time windows of pregnancy or throughout gestation, may reflect dynamic changes of mitochondrial function, and thus, be associated with suboptimal neonatal anthropometric measures. In the National Institute of Child Health and Human Development (NICHD) Fetal Growth Studies-Singletons, we specifically examined the longitudinal and prospective association of acylcarnitines throughout pregnancy with neonatal anthropometrics, including birthweight, birthweight z score, birth length, sum of skinfolds, and sum of body circumferences. Moreover, we examined the potential effect modification by the maternal physiological status (i.e., obesity and gestational diabetes), race/ethnicity, and infant sex.

## 2. Results

Baseline characteristics of the participants in the final analysis are shown as median (IQR) or frequency (percentages) (Table 1). Over the entire gestational period, median levels of 22 individual acylcarnitines differed to varying extents. For each acylcarnitine, its distributions were very similar across four visits (data not shown). There were few statistically significant differences in individual acylcarnitines between women with and without obesity at any study visit. Women with obesity had significantly higher C4 levels at GW 10–14, lower C5 at GW 15–26, and C14:OH at GW 1–14 than those without obesity. 

### 2.1. Associations between Acylcarnitines and Neonatal Biometry in Each Visit

**Figure 1:**
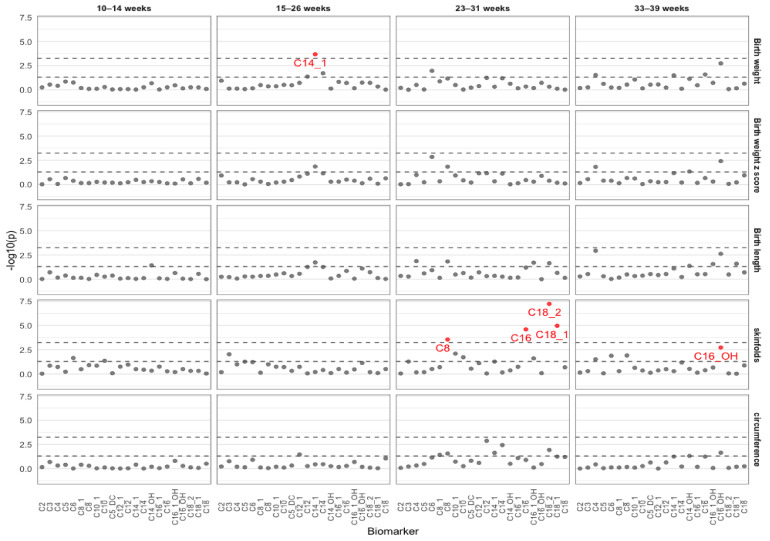
Manhattan plot for individual acylcarnitines in association with neonatal birthweight, birthweight z score, birth length, sum of skinfolds, and sum of circumferences. The lower dash line denotes the nominal *p*-value of 0.05, the upper dash line corresponds to the Bonferroni threshold of 0.05/88, and the *p* values that are FDR significant are colored red.

Most of the individual acylcartinines (17/22; 77%) were significantly associated with birthweight, birthweight z score, birth length, sum of skinfolds or sum of circumferences for at least one visit (Figure 1 and Supplementary Table S1). C12, C14, C14:1 at GW15–26, C6 at GW23–31, and C4 and C16 at GW33–39 were inversely associated with birthweight (as a continuous outcome in either gram or a z score), while C8 at GW23–31, and C14-OH and C16-OH at GW33–39 were positively associated with birthweight (either in gram or z score). For birth length, the metabolites with inverse associations included C4 at GW23–31 and GW33–39, C14:1 at GW15–26, C16:1–OH and C18:2 at GW23–31, while those with positive associations included C8 at GW23–31, C14–OH at GW10–14, and GW33–39, C16-OH, C16:1-OH, and C18:1 at GW33–39. Furthermore, we identified 11 acylcarnitines that were significantly associated with the sum of skinfolds, six of which showed an inverse association, while the other five showed a positive association. Specifically, higher levels of C3 at GW15–26, C8 at GW23–31 and GW33–39, C10 at GW23–31, C10:1 at GW23–31, and C16-OH at GW33–39 were associated with thicker skinfolds, while higher levels of C4 at GW33–39, C16 and C16:1OH at GW23–31, and C18:1 and C18:2 at GW23–31 were associated with thinner skinfolds. Interestingly, the relation between C6 and the sum of skinfolds was non-linear. C6 at GW10–14 was significantly and positively associated with skinfolds, whereas C6 at GW33–39 was inversely associated with the sum of skinfolds. Nine out of 22 acylcartinines (41%) were significantly associated with the sum of circumferences. We observed positive associations for C8, C12, C14, C8:1, C14:1, and C18:2 at GW23–31, as well as C14-OH and C16-OH at GW33–39 and inverse associations for only C12:1 at GW15–26 with the sum of circumferences.

### 2.2. Longitudinal Associations between Individual Acylcarnitine Trajectories and Neonatal Biometry

Longitudinally, each acylcarnitine had one or more distinct trajectory groups across gestation (Supplementary Figure S1). Of the 22 acylcarnitines in the group-based trajectory analysis, we identified three distinct subgroups for C2, C3, and C4; two groups for C5, C8:1, C10:1, C10, C5:DC, and C12; and only one group for all others across gestation. Having one trajectory subgroup is the most frequent pattern in 13 acylcarnitines (59%), in which all of the women had similar longitudinal change patterns. Of them, the levels of C6, C8, C14, C16, C18:2, C18:1, and C18 decreased over pregnancy, whereas C12:1, C14:1, and C16:1-OH increased. In addition, levels of C14-OH, C16:1, and C16-OH remained unchanged during pregnancy. We identified C10, C10:1, and C12 that had significant group-differences in at least one of neonatal anthropometric measures. The first trajectory group of C12 (94.3%) remained stable during gestation, while the second trajectory group of C12 (5.66%) varied widely, with a slightly increasing trend early in pregnancy (prior to 18 weeks) followed by a significantly decreasing trend towards the end of pregnancy. Women in the second trajectory group of C12 had significantly lower levels of both birthweight and birth length than those in the first stable trajectory group of C12. Two trajectory groups were identified in C10, both of which showed decreasing but nearly parallel trends during pregnancy. Women with persistently higher levels of C10 (6.07%) had larger skinfolds than those with lower levels (93.9%) (Table 2). Levels of C10:1 varied appreciably between two trajectory groups across pregnancy: One with an increasing nonlinear trend, and another with a decreasing linear trend. Women categorized in the trajectory with an abruptly increased trend of C10:1 (87.4%) starting from the mid-trimester had a larger sum of skinfolds than those categorized in the trajectory with a slightly decreased trend (12.6%) (Table 2).

### 2.3. Associations of Joint Acylcarnitine Trajectories and Neonatal Biometry

Using K-means clustering, the dynamic changes in all of the 22 acylcarnitines were significantly stratified into two clusters (cluster A and B in 72% (*n* = 231) and 28% (*n* = 90) of all participants, respectively). When plotting the two clusters identified from joint trajectory analyses (in 2D for each acylcarnitine), there were no significant differences in the trajectory patterns of each acylcarnitine during pregnancy (Supplementary Figure S2). The association analyses between the clustering groups with respect to anthropometric outcomes suggested that the overall trajectory profile of all the 22 acylcartinines during pregnancy was not significantly associated with neonatal anthropometry (Supplementary Table S2).

### 2.4. Lack of Significant Effect Modifications by Fasting Status, GDM, and Infant Sex

In the sensitivity analyses, we identified a significant interaction between C14:1 at weeks 15–26 and time since the last meal on birthweight but not on birthweight z score, indicating that the overall impact of fasting status in plasma acylcarnitines was either absent or inconsequential (data not shown). Similarly, the results did not materially change when we excluded individuals with non-fasting plasma samples. In addition, neither the GDM status nor infant sex substantially changed the significant results of C10, C10:1, and C12 with longitudinal associations with at least one of the neonatal anthropometric measures (data not shown).

## 3. Discussion

In this longitudinal study, we investigated 22 aclycartinines with discernible plasma levels during gestation, most of which were significantly associated with birthweight, birth length, sum of skinfolds or sum of body circumferences in at least one visit. Longitudinally, distinct trajectories of three medium-chain acylcartinines—namely, C12, C10, and C10:1—have a significant linear or nonlinear association with neonatal anthropometric measures, while plasma levels of all acylcartinines varied substantially during pregnancy.

While it is increasingly evident that the optimal mitochondrial function of different tissues and organs is essential to meet the nutritional and metabolic demands of both the mother and developing fetus, characterizing mitochondrial function in the context of pregnancy is particularly important. Circulatory acylcarnitines have been used as biomarkers of systemic mitochondrial dysfunction, reflective of accumulating acyl-CoA metabolites in cells under conditions, such as a mismatch between FAO and glucose oxidation [8,11]. The human placenta has an extensive ability to uptake lipid species and shuttle them and their metabolic byproducts into fetal circulation [19]. In the placenta, acylcarnitines can facilitate the transport of long-chain fatty acids into the mitochondria where fatty acids undergo β-oxidation [4,19]. In addition, individual acylcarnitines can directly activate classical proinflammatory signaling pathways [20], ultimately inducing placental mitochondrial dysfunction related to adverse pregnancy complications. In this regard, we hypothesized that altered acylcarnitine levels in maternal circulation are indicators of maternal mitochondrial function, which is associated with placental mitochondrial function and subsequent fetal growth.

The introduction and advancement of tandem mass spectrometry technology enables the quantification of plasma acylcarnitine profiles in population studies. This study was a comprehensive exploration of acylcarnitine profiles during pregnancy in relation to neonatal anthropometry since we applied a targeted LC-MS/MS method to ascertain a panel of acylcarnitine species, including other types of acylcarnitines, such as unsaturated, dicarboxylic acids (C5-DC), and hydroxylated acids (-OH), at four time points throughout pregnancy.

Despite limited longitudinal data, prior studies have suggested time-dependent fluctuations in levels of specific acylcarnitines during pregnancy with heterogenous trajectory patterns [21,22,23]. Whether dynamic changes in acylcarnitines are related to fetal outcomes had remained unclear, given the limited longitudinal data. Specifically, our results highlight the significant associations between trajectory changes in acylcarnitines, particularly two medium-chain saturated acylcarnitines (C12 and C10) and one medium-chain monounsaturated acylcarnitine (C10:1), and multiple neonatal anthropometric measures. These significant associations persisted after adjustment for confounding variables and correction for multiple comparisons.

Recently, a multi-ethnic cohort of mother-newborn dyads, the Hyperglycemia and Adverse Pregnancy Outcome (HAPO) study, profiled the maternal metabolome using targeted (63 metabolites) and non-targeted (76 metabolites) metabolomics in serum samples at GW24–32 [15,18]. The HAPO study reported some of the same patterns we observed in the current study. In an earlier report from the HAPO study of 400 mothers of European ancestry, maternal C8 and C10 levels were inversely associated, but C10:3 was positively associated, with birthweight and skinfolds [15]. That study also found other acylcarnitines with varying lengths, such as C8, C8:1, and C10:3, and several acylcarnitines with OH and DC to be associated with newborn birthweight and skinfolds. Furthermore, a recent report from the HAPO study was extended to a total of 1600 mothers in four ancestry groups (Northern European, Afro-Caribbean, Mexican American, and Thai) [18]. Similar to our significant findings, maternal C10 and C12 levels were associated with newborn skinfolds independent of maternal BMI and glucose levels [18].

Our significant findings were also consistent with those from four European birth cohorts applying untargeted metabolomics to 481 cord blood samples collected at delivery [24]. They found a significant association between lower levels of acylcarnitine species (C4, C6, C8, C10, C12, C14, and C16) and increased birth weight [24]. However, the correlation between maternal and cord blood metabolites is generally high, but may vary depending on the metabolites [25]. It is also worth mentioning that these studies lacked longitudinal measurements of acylcarnitines from early pregnancy and failed to provide complete information on time-integrated acylcarnitine profiling across pregnancy in association with neonatal anthropometric measures.

In contrast, a few studies reported no significant associations between acylcarnitines and neonatal adiposity, especially after correction for multiple testing and adjustment for confounders [26,27]. These discrepant findings may be due to the fact that these studies varied in several aspects, including the timing of blood sampling (early, mid or late gestation), number of participants, sample type, fasting status, and selection of metabolomics assessment approaches targeting different classes of metabolites, including acylcarnitines. We cannot rule out the possibility that the limited variation of some acylcarnitines may limit their statistical power to identify significant associations with neonatal anthropometric measures. Additionally, the characteristics of the study populations, including ethnic ancestry, maternal BMI, weight gain during pregnancy, lipid status, GDM, and glycemic control status may explain, in part, the inconsistent results in population studies.

In line with previous studies [15,18,28], we observed varying results for some, but not all acylcarnitines between obese women and non-obese women and between GDM cases and non-GDM women. However, our sensitivity analyses showed that the associations of C12, C10, and C10:1 with neonatal anthropometric measures remained significant even after adjustment for GDM and pre-pregnancy BMI. Additionally, several other factors are also likely to affect maternal metabolic profiles, such as gestational age, race/ethnicity, infant sex, and fasting status of blood samples. While it is possible that plasma levels of acylcarnitines are sensitive to the fasting state, our sensitivity analyses found C12, C10, and C10:1 are relatively stable regardless of fasting. Taken together, our findings of associations between medium-chain acylcarnitines and neonatal anthropometry tend to be robust, independent of maternal glycemic and adiposity status, although further replication and prediction investigations are required to confirm or refute our findings.

A major strength of our study is the longitudinal measurement of plasma levels of acylcarnitines at four time points throughout pregnancy, which gave us the opportunity to examine the temporal relationship between longitudinal changes of acylcarnitines during pregnancy and neonatal anthropometry. In particular, we applied targeted LC-MS/MS to interrogate a comprehensive panel of 22 acylcarnitine species, including unsaturated, hydroxyl, and dicarboxylic carnitine derivatives, and compare the strength of their associations with our outcomes. The race/ethnic diversity of the study cohort increased the generalizability of our results.

However, some potential limitations need to be acknowledged. First, the relatively small sample size limited our ability to address racial/ethnic disparities in the relationship between acylcarnitines and neonatal anthropometry, and we were only able to explore the possible effect modification of these associations by broad categorization of race/ethnicity. Second, we applied k-means based approaches to explore the clustering of acylcarnitine metabolites in association with neonatal outcomes. These data-driven algorithms are based on the statistical properties of the underlying data structure rather than previous knowledge or biology-based classifications. Third, concomitant changes in metabolites other than acylcarnitines, such as branched-chain and aromatic amino acids, non-esterified fatty acids, and phosphatidylcholines, might induce collinearity and further complicate the associations of acylcarnitines, which is difficult to disentangle, especially when they might be highly correlated or in the same biological pathways. We previously reported that most of the maternal acylcarnitines (except C8 and C14) appeared to significantly decline in early/mid-pregnancy and rise slightly after GW30, while the sum of BCAA significantly decreased as gestation progressed, driven by changes in leucine and valine [28]. Finally, the results do not prove a causal effect of acylcarnitines on fetal growth and development.

## 4. Materials and Methods

### 4.1. Study Design and Population

We used data from a nested case-control study within the Eunice Kennedy Shriver NICHD Fetal Growth Studies-Singleton cohort (2009–2013). The full cohort consisted of 2802 generally healthy women (2334 non-obese and 468 obese women) with singleton pregnancies and aged 18–40 years at enrollment [29,30]. All of the women were enrolled between 8 weeks 0 days and 13 weeks 6 days of gestation at 12 clinical centers throughout the US and were followed throughout their pregnancies [29,30]. For participants to be eligible, ultrasound estimates of gestational age at enrollment were required to be consistent (±5–7 days) with gestational dating, calculated by the last menstrual period. Sampling and eligibility criteria are described in detail elsewhere [29,30]. The study was approved by all of the participating institutions including NICHD. All of the study participants gave their written informed consent prior to enrollment.

In the nested case-control study, a total of 107 women with incident GDM were identified as cases and matched randomly at a ratio of 1:2 to non-GDM controls on age (±2 years), race/ethnicity (non-Hispanic white, non-Hispanic black, Hispanic or Asian/Pacific Islander), and gestational age at blood collection (±2 weeks) [31,32]. The screening or diagnosis of GDM was conducted according to the standard clinical care, at an average gestational age of 27 weeks [33]. Finally, we included 321 women with plasma samples in our analysis (107 women with GDM and 214 women without GDM).

### 4.2. Assessment of Acylcarnitine Profiling

In this prospective cohort study, maternal blood samples were longitudinally collected from each participant at four study visits during pregnancy: Gestational weeks 8–13 (enrollment visit), 16–22 (visit 1), 24–29 (visit 2), and 34–37 (visit 4) [29,30]. However, the actual time ranges for blood collection were gestational weeks 10–14, 15–26, 23–31, and 33–39, respectively. All of the biospecimens were processed immediately and stored at −80 °C before the assay [29,30]. All of the women were instructed to fast overnight for 8–14 h before their blood samples were drawn at weeks 15–26.

We adopted a previously published protocol to measure acylcarnitine species in plasma using electrospray ionization tandem mass spectrometry (ESI–MS/MS) in plasma [10]. Short-, medium-, and long-chain acylcarnitines were defined as having carbon chains ≤6, 8–14, and ≥16 in length, respectively. As shown in Table 3, 28 acylcarnitines (µmol/L) were quantified as follows: 1) Short-chain acylcarnitines: C2 (Acetylcarnitine), C3 (Propionylcarnitine), C4 (Butyrylcarnitine), C5 (Valerylcarnitine), and C6 (Hexanoylcarnitine); 2) medium-chain acylcarnitines: C8 (Octanoylcarnitine), C8:1 (Octenoylcarnitine), C10 (Decanoylcarnitine), C10:1 (Decenoylcarnitine), C12 (Dodecanoylcarnitine), C12:1 (Dodecenoylcarnitine), C14 (Tetradecanoylcarnitine), C14:1 (Tetradecenoylcarnitine), and C14:2 (Tetradecadienylcarnitine); 3) long-chain acylcarnitines: C16 (Hexadecanoylcarnitine), C16:1 (Hexadecenoylcarnitine), C18 (Octadecanoylcarnitine), C18:1 (Octadecenoylcarnitine), and C18:2 (Octadecadienylcarnitine); 4) carnitine esters derived from dicarboxylic acids: C5-DC (Glutarylcarnitine); and 5) carnitine esters derived from hydroxylated acids: C6-OH (3-OH-Hexanoylcarnitine), C12-OH (3-OH-Dodecanoylcarnitine), C14:1-OH (3-OH-Tetradecenoylcarnitine), C14-OH (3-OH-Tetradecanolycarnitine), C16:1-OH (3-OH-Hexadecenoylcarnitine), C16-OH (3-OH-Hexadecanoylcarnitine), C18:1-OH (3-OH-octadecenoylcarnitine), and C18:2-OH (3-OH-Octadecadienoylcarnitine).

The analysis was performed at Mayo Medical Laboratories. Briefly, six internal standards of known concentrations were added to the plasma sample: D3-acetylcarnitine, D3-propionylcarnitine, D7-butyrylcarnitine, D3-octanoylcarnitine, D3-odecanoylcarnitine, and D3-palmitoylcarnitine. Samples were deproteinized with the addition of acetonitrile. Following shaking and centrifugation, the supernatants were dried and derivatized with n-butanolic HCl, yielding acylcarnitines in n-butyl-ester form. Then, the samples were resuspended in a running buffer and analyzed by ESI–MS/MS [9,10]. The concentrations of the analytes were estimated by a computerized comparison of their ion intensities to internal standards. It should be noted that Mayo Medical Laboratories used the flow injection analysis in order that the C5-DC butyl ester peak was the sum of both C5-DC and C10-OH butyl ester.

### 4.3. Neonatal Anthropometry

As described previously [34], birthweight and gestational age at delivery were abstracted from neonatal medical records. Gestational age- and sex-specific birthweight z scores were derived using an US national reference [35]. Neonatal length and skinfolds were measured by trained, certified study personnel. Measurements were obtained prior to discharge, 12–24 h after delivery. When it was impossible to obtain measurements at birth due to the NICU admission or neonatal complications, infants born very preterm (≤ 32 weeks) were measured at 32 completed weeks of gestation-corrected age, and infants born moderately preterm (33–36 weeks) were measured once stabilized. Measurements were obtained in duplicate and a third measurement was obtained if the difference between the first two measurements exceeded the prespecified tolerances based on expected technical errors of measurement [36,37,38]. Two closest measurements were averaged. Neonatal length, i.e., distance from soles of infant’s feet to the top of the head, was measured with infant supine using an Infantometer (SECA 416 Infantometer) (SECA, Chino, CA, USA). Skinfold measurements were taken on the right side of the infant’s body at the abdominal flank, anterior thigh, subscapular, and triceps using a Lange Skinfold Caliper (Beta Technology, Inc., Santa Cruz, CA, USA) and summed as an indicator for total adiposity [39,40]. Circumference measurements were taken on neonatal head, chest, abdominal (at a level midway between the xiphoid process and the umbilicus), umbilical, and mid-upper arm and summed for body circumferences [41].

### 4.4. Assessment of Covariates

Information on participant demographics, lifestyle factors, and past medical history was collected through a self-reported questionnaire. A priori selection of covariates, including nulliparity (yes/no) and pre-pregnancy BMI (kg/m^2^), were assessed at study enrollment. Given that cases were matched with controls within a certain range of maternal age (years) and gestational age at biospecimen collection (weeks), we also included these two matching variables as covariates in the analysis.

### 4.5. Statistical Analysis

We identified 28 known acylcarnitines in the samples. Among the 28 identified acylcarnitines, six were excluded from the final data analysis due to the reason that over 30% of the measurements were below the LOD (C12, C14:2, C18:2, and C18:1) or with no variability (C6:OH and C14:1-OH). For the remaining 22 acylcarnitine species in the final analysis, we imputed all of the non-detections to values of LOD/square root of 2. Spearman’s rank-order correlation was used to calculate correlation coefficients among acylcarnitines at each visit.

For individual acylcarnitines across the four visits during gestation in association with neonatal anthropometric indices, we performed multivariable linear regression models with robust standard errors accounting for sampling weights. Since women with GDM were overrepresented in the analytic sample with biomarkers, the sample was reweighed to represent the full cohort (e.g., in the reweighed sample 4% of women had GDM as opposed to 33% in the nonweighed sample). As described previously [31,42], weights were created per each subject by the inverse of her sampling probability [34]. The sampling probability of each non-GDM subject was calculated from a logistic regression in the full cohort, excluding GDM cases. Predictors included matching factors for selecting controls: Age, race/ethnicity, and gestational week at blood collection.

Each individual acylcarnitine was modeled as a continuous variable (per 1 SD increase) at each of the four visits during gestation. Regression coefficients from the generalized linear model (GLM) were obtained for neonatal anthropometric measures (continuous) per SD increase in individual acylcarnitine. The main multivariable linear models were adjusted for maternal age (continuous), race/ethnicity (non-Hispanic white, non-Hispanic black, Hispanic, Asian/Pacific Islander), education (High school or less, some college/associate degree, 4-year college degree or higher), nulliparity (yes/no), pre-pregnancy BMI (continuous), gestational age at blood collection (continuous), and postnatal days at neonatal assessment (continuous; for length, skinfolds, and circumference models only). Multiple testing was corrected using the Benjamini–Hochberg false discovery rate (FDR) procedure [43].

To characterize how individual acylcarnitine changed over gestation, we further applied a flexible data-driven semiparametric approach to identify longitudinal trajectories across four visits [44]. We compared the model fit (linear, quadratic, and cubic) for each acylcarnitine biomarker and chose the best model fit based on Jeffreys’s scale [44]. We chose final models that had ≥5% of the data in each identified group. The between-group (trajectory) difference in neonatal anthropometry for each metabolite was obtained using a weighted multivariate linear regression model with robust standard errors, adjusting for the aforementioned confounders for the associations of individual acylcarnitine.

To visualize and identify potential patterns of acylcarnitine clustering during gestation, we adopted the k-means clustering analysis to study the joint evolution of 22 biomarker trajectories over four visits. The Calinski and Harabasz criterion was used to determine the optimal number of clusters [45]. As a result, the model with two clusters showed the best performance. Subsequently, we included the clusters in multivariable-adjusted linear regression analyses to assess whether the between-cluster difference was statistically associated with each of the five neonatal anthropometric measures, respectively.

We conducted sensitivity analyses to assess the robustness of our findings. First, to evaluate the effect of timing of blood draw, we tested the interaction by a variable of time since the last meal and also performed the complete data analyses among all of the individuals with fasting plasma samples. Second, to assess whether GDM, which has been associated with alterations in the metabolome, influenced the results, we compared the results of subgroup analysis stratified by GDM. Furthermore, to explore the possible effect of modification in the relationship between acylcarnitines and neonatal outcomes, we performed subgroup analyses stratified by race/ethnicity (non-Hispanic White vs. all others), GDM (yes or no), pre-pregnancy BMI status (normal weight or overweight/obese), and infant sex (male or female).

All of the analyses incorporated the original study design and sampling weights, except for the k-means clustering analysis. Statistical significance was set at FDR < 0.05. Unless otherwise specified, statistical analyses were performed using the SAS software (version 9.4, SAS Institute, Cary, NC) and R3.4.3. The K-means clustering of all the acylcarnitines was calculated in the R software (R 3.4.2 version; Boston, MA, USA).

## 5. Conclusions

In conclusion, we found that the gestational trajectories of C10, C10:1, and C12 acylcarnitine levels are significantly associated with neonatal anthropometry. Our results indicate the critical importance of some medium-chain acylcarnitines as markers of systemic maternal mitochondrial function, predictive of fetal growth and development. Further longitudinal investigation is warranted to replicate our findings and evaluate clinical usefulness.

## Figures and Tables

**Table 1:** Baseline characteristics of study participants with GDM and their age- and race-matched controls in the NICHD Fetal Growth Studies-Singleton Cohort.

Characteristics	Participants (*n* = 321) ^a,b^
Age, years	28.0 (24.0, 32.0)
Race-ethnicity	
Non-Hispanic White	75 (30.9)
Non-Hispanic Black	45 (23.3)
Hispanic	123 (27.2)
Asian and Pacific Islander	78 (18.5)
Education	
High school or less	81 (25.1)
Some college/associate degree	117 (35.2)
Four-year college degree or higher	123 (39.8)
Nulliparous	143 (51.1)
Smoked	5 (0.7)
Pre-pregnancy BMI, kg/m2 (self-reported)	24.6 (22.0, 27.5)
19.0–24.9	156 (51.73)
25.0–29.9	99 (33.05)
30.0–45.0	66 (13.22)
Gestational diabetes	107 (3.9)
Infant sex	
Male	166 (52.04)
Female	153 (47.96)

^a^ Continuous and categorical variables were summarized using weighted median (IQR) or counts (weighted percentage), respectively; ^b^ *p* values for group comparisons were calculated using the chi-square test considering weights for categorical variables, and the Wilcoxon test considering weights for continuous variables.

**Table 2:** Acylcarnitine trajectories across gestation and the association with neonatal birthweight, birthweight z score, length, sum of skinfolds, and sum of circumstances ^a,b^.

Maternal Acylcarnitines	Neonatal Outcome, Adjusted β (95% CI)				
	Birthweight, g	Birthweight, z Score	Length, cm	Sum of Skinfolds, mm	Sum of Circumference, cm
C2					
Group 3	−59.3 (−521, 403)	0.11 (−0.41, 0.64)	0.57 (−1.61, 2.74)	2.01 (−2.58, 6.60)	−0.46 (−6.11, 5.19)
Group 2	85.6 (−157, 329)	0.11 (−0.25, 0.47)	0.32 (−0.77, 1.41)	0.84 (−1.88, 3.55)	0.14 (−4.27, 4.55)
Group 1	0.0 (ref)	0.0 (ref)	0.0 (ref)	0.0 (ref)	0.0 (ref)
C3					
Group 3	188 (−113, 490)	0.17 (−0.29, 0.62)	1.13 (−0.44, 2.70)	−0.92 (−4.03, 2.20)	2.92 (−2.01, 7.85)
Group 2	115 (−55.5, 286)	0.26 (−0.08, 0.60)	0.30 (−0.90, 1.50)	−1.12 (−3.74, 1.51)	2.24 (−1.65, 6.14)
Group 1	0.0 (ref)	0.0 (ref)	0.0 (ref)	0.0 (ref)	0.0 (ref)
C4					
Group 3	44.2 (−187, 275)	−0.13 (−0.51, 0.25)	−0.42 (−2.08, 1.24)	−0.47 (−1.89, 0.94)	1.77 (−2.60, 6.14)
Group 2	80.9 (−107, 269)	−0.03 (−0.29, 0.22)	0.29 (−0.68, 1.26)	0.46 (−1.24, 2.15)	0.10 (−2.77, 2.97)
Group 1	0.0 (ref)	0.0 (ref)	0.0 (ref)	0.0 (ref)	0.0 (ref)
C5					
Group 2	−86.3 (−324, 151)	−0.25 (−0.57, 0.07)	−0.10 (−1.56, 1.37)	0.17 (−1.89, 2.24)	−1.34 (−5.84, 3.16)
Group 1	0.0 (ref)	0.0 (ref)	0.0 (ref)	0.0 (ref)	0.0 (ref)
C8:1					
Group 2	−159 (−414, 95.3)	−0.17 (−0.47, 0.14)	−0.12 (−1.39, 1.15)	−1.37 (−3.64, 0.90)	−2.01 (−5.58, 1.55)
Group 1	0.0 (ref)	0.0 (ref)	0.0 (ref)	0.0 (ref)	0.0 (ref)
C10:1					
Group 2	−127 (−508, 253)	−0.05 (−0.39, 0.30)	−0.26 (−1.95, 1.43)	3.23 (0.19, 6.27)	−0.95 (−4.54, 2.65)
Group 1	0.0 (ref)	0.0 (ref)	0.0 (ref)	0.0 (ref)	0.0 (ref)
C10					
Group 2	31.4 (−165, 227)	−0.08 (−0.45, 0.29)	−0.28 (−1.33, 0.77)	4.91 (0.85, 8.98)	−2.64 (−8.39, 3.11)
Group 1	0.0 (ref)	0.0 (ref)	0.0 (ref)	0.0 (ref)	0.0 (ref)
C5:DC					
Group 2	−500 (−1104, 104)	−0.34 (−0.82, 0.14)	−1.31 (−2.99, 0.36)	−1.50 (−3.84, 0.84)	−1.61 (−6.94, 3.72)
Group 1	0.0 (ref)	0.0 (ref)	0.0 (ref)	0.0 (ref)	0.0 (ref)
C12					
Group 2	−475 (−942, −6.79)	−0.39 (−0.71, −0.06)	−1.38 (−2.49, −0.27)	−2.00 (−5.06, 1.07)	−1.90 (−6.69, 2.88)
Group 1	0.0 (ref)	0.0 (ref)	0.0 (ref)	0.0 (ref)	0.0 (ref)

^a^ Those with only one identified trajectory group are not included; ^b^ each trajectory group was then associated with each neonatal outcome using a weighted multivariate regression, adjusting for age, pre-pregnancy BMI, race-ethnicity, education, nulliparity.

**Table 3:** Median and interquartile range (IQR) of 22 acylcarnitines (µmol/L) by four visits.

Groups	Acylcarnitines	Gestational Weeks			
		10–14	15–26	23–31	33–39
Short-chain	C2 (Acetylcarnitine)	3.15 (2.59, 3.99)	2.94 (2.28, 3.73)	2.56 (1.95, 3.12)	2.60 (2.01, 3.05)
	C3 (Propionylcarnitine)	0.21 (0.15, 0.25)	0.16 (0.13, 0.19)	0.17 (0.13, 0.21)	0.16 (0.12, 0.20)
	C4 (Butyrylcarnitine)	0.15 (0.12, 0.20)	0.12 (0.09, 0.15)	0.13 (0.09, 0.17)	0.13 (0.09,0.15)
	C5 (Valerylcarnitine)	0.06 (0.04, 0.08)	0.05 (0.04, 0.06)	0.05 (0.04, 0.07)	0.06 (0.04, 0.07)
	C6 (Hexanoylcarnitine)	0.02 (0.01, 0.03)	0.02 (0.01, 0.03)	0.01 (0.01, 0.02)	0.01 (0.01, 0.02)
Medium-chain	C8 (Octanoylcarnitine)	0.08 (0.05, 0.11)	0.10 (0.08, 0.12)	0.07 (0.05, 0.09)	0.08 (0.05, 0.10)
	C8:1 (Octenoylcarnitine)	0.07 (0.06, 0.11)	0.08 (0.05, 0.11)	0.07 (0.04, 0.10)	0.07 (0.04, 0.09)
	C10 (Decanoylcarnitine)	0.06 (0.03, 0.10)	0.08 (0.05, 0.11)	0.04 (0.02, 0.06)	0.05 (0.03, 0.07)
	C10:1 (Decenoylcarnitine)	0.05 (0.03, 0.08)	0.07 (0.05, 0.08)	0.04 (0.03, 0.06)	0.04 (0.03, 0.07)
	C12 (Dodecanoylcarnitine)	0.03 (0.02, 0.04)	0.03 (0.02, 0.04)	0.02 (0.02, 0.03)	0.02 (0.02, 0.03)
	C12:1 (Dodecenoylcarnitine)	0.02 (0.01, 0.03)	0.02 (0.01, 0.03)	0.01 (0.01, 0.02)	0.02 (0.01, 0.02)
	C14 (Tetradecanoylcarnitine)	0.01 (0.01, 0.02)	0.01 (0.01, 0.02)	0.01 (0.01, 0.02)	0.01 (0.01, 0.02)
	C14:1 (Tetradecenoylcarnitine)	0.02 (0.01, 0.03)	0.02 (0.01, 0.03)	0.01 (0.01, 0.02)	0.01 (0.01, 0.02)
Long-chain	C16 (Hexadecanoylcarnitine)	0.05 (0.04, 0.06)	0.05 (0.04, 0.06)	0.04 (0.04, 0.05)	0.04 (0.03, 0.06)
	C16:1 (Hexadecenoylcarnitine)	0.01 (0.01, 0.01)	0.01 (0.01, 0.01)	0.01 (0.007, 0.01)	0.01 (0.01, 0.01)
	C18 (Octadecanoylcarnitine);	0.02 (0.02, 0.03)	0.02 (0.02, 0.03)	0.02 (0.02, 0.03)	0.02 (0.02, 0.03)
	C18:1 (Octadecenoylcarnitine)	0.05 (0.04, 0.08)	0.05 (0.04, 0.06)	0.04 (0.03, 0.06)	0.04 (0.03, 0.06)
	C18:2 (Octadecadienylcarnitine)	0.03 (0.02, 0.03)	0.02 (0.02, 0.03)	0.02 (0.01, 0.02)	0.02 (0.01, 0.02)
Carnitine esters derived from dicarboxylic acids	C5-DC (Glutarylcarnitine)	0.02 (0.01, 0.02)	0.02 (0.01, 0.02)	0.01 (0.01, 0.02)	0.01 (0.01, 0.02)
Carnitine esters derived from hydroxylated acids	C14-OH (3-OH-Tetradecanolycarnitine)	0.000 (0.000, 0.02)	0.000 (0.000, 0.01)	0.000 (0.000, 0.02)	0.000 (0.000, 0.01)
	C16:1-OH (3-OH-Hexadecenoylcarnitine)	0.000 (0.000, 0.04)	0.000 (0.000, 0.01)	0.000 (0.000, 0.03)	0.000 (0.000, 0.01)
	C16-OH (3-OH-Hexadecanoylcarnitine)	0.000 (0.000, 0.01)	0.000 (0.000, 0.02)	0.000 (0.000, 0.03)	0.000 (0.000, 0.02)

## Data Availability

Data described in the manuscript, code book, and analytic code will be made available upon request pending review and approval by the Institutional Review Board.

## Supplementary Materials

The following are available online at www.mdpi.com/article/10.3390/metabo11120885/s1. Figure S1: Acylcarnitine trajectory across gestational weeks, Figure S2: Joint trajectory of acylcarnitine across gestational week, Table S1: Acylcarnitine in association with neonatal birthweight, birthweight z score, length, sum of skinfolds, and sum of body circumstance, Table S2: Joint trajectory of acylcarnitines across gestation and the association with neonatal birthweight, birthweight z score, length, sum of skinfolds, and sum of body circumstances.
