# Supplementary material for: Plasma Acylcarnitines during Pregnancy and Neonatal Anthropometry: A Longitudinal Study in a Multiracial Cohort
Source: Metabolites. 2021 Dec 17;11(12):885. doi: 10.3390/metabo11120885 (PMC8704426; doi:10.3390/metabo11120885)
Supplement: Supplementary file 1 [file metabolites-11-00885-s001.zip › metabolites-1468536-supplementary.pdf]

Supplementary Figure S1. Acylcarnitine trajectory across gestational weeks [a,b]

C2	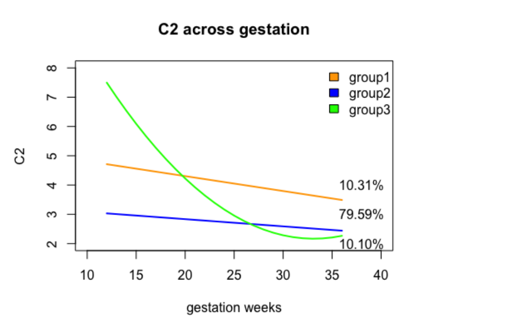	C3	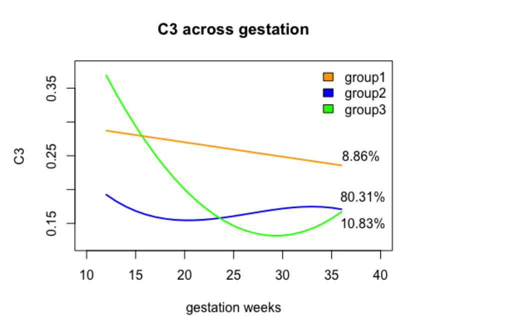
C4	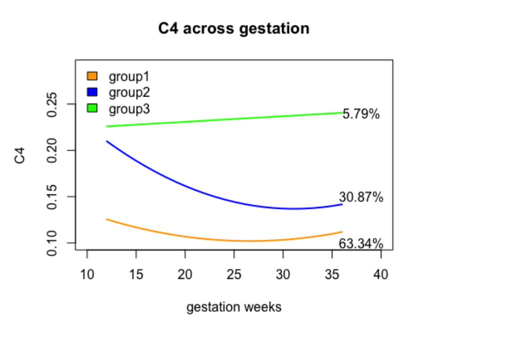	C5	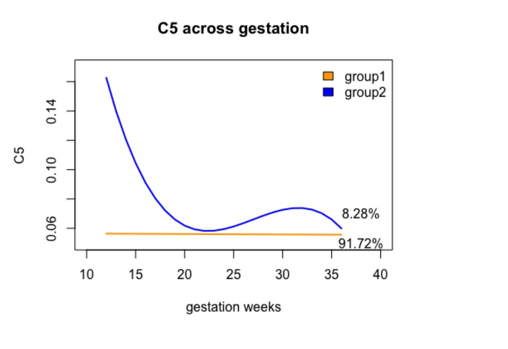
C6	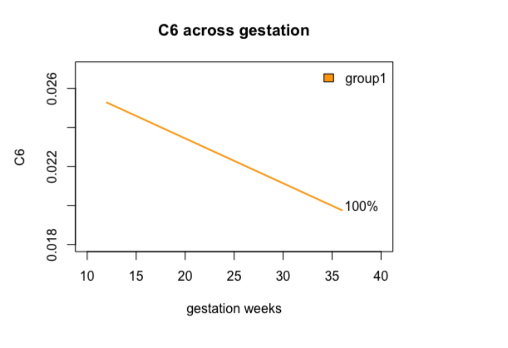	C8:1	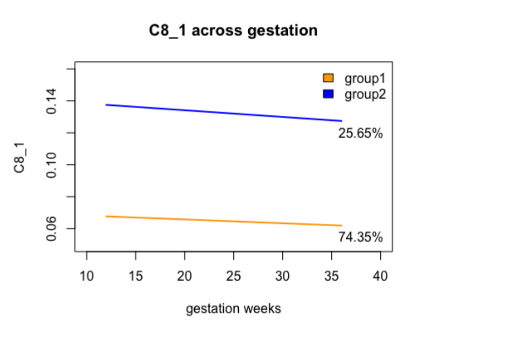
C8	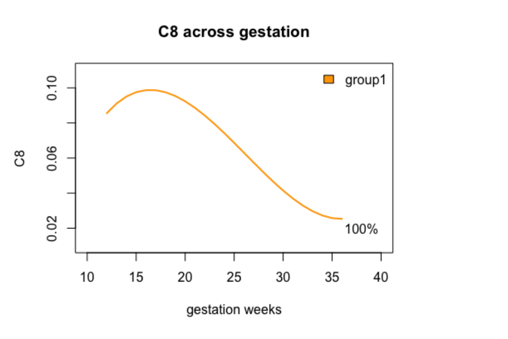	C10:1	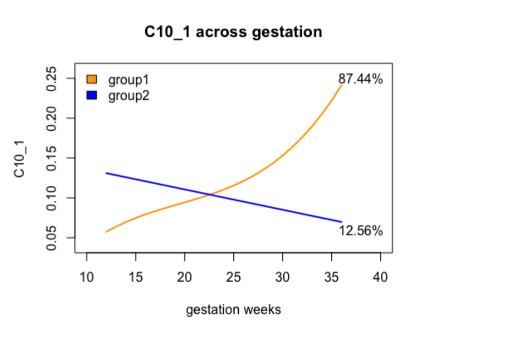
C10	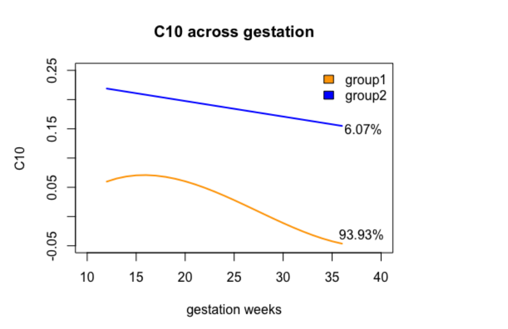	C5:DC	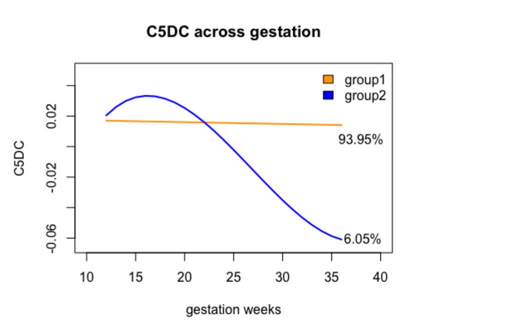

C12:1	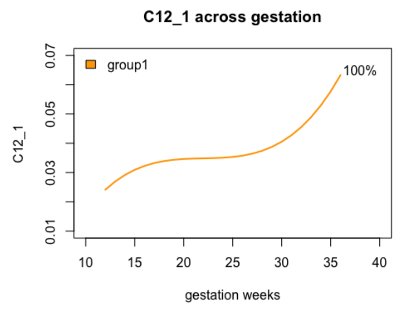	C12	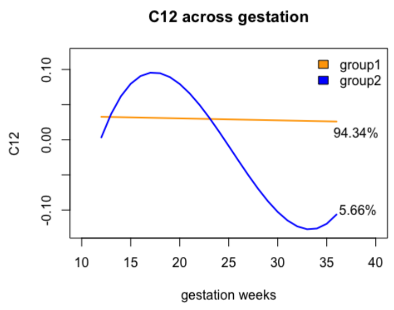
C14:1	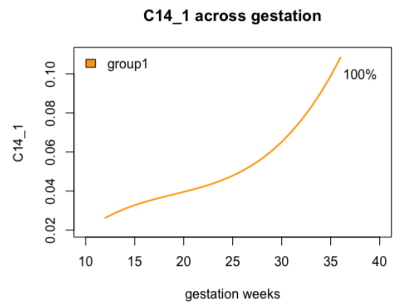	C14	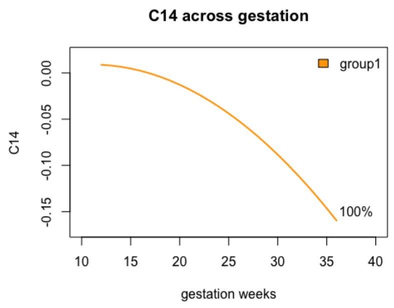
C14_OH	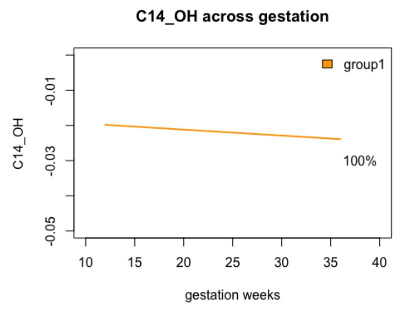	C16_1	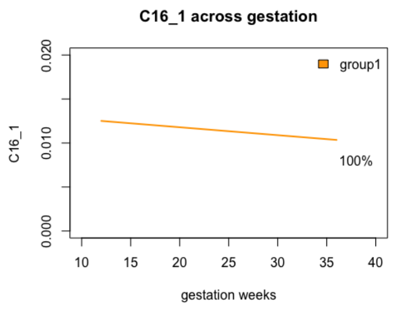
C16	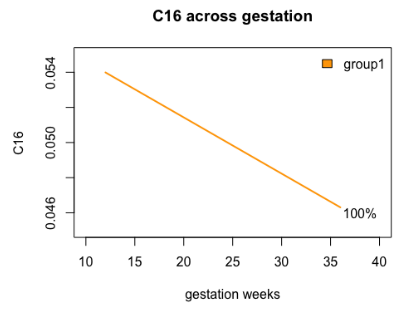	C16_1OH	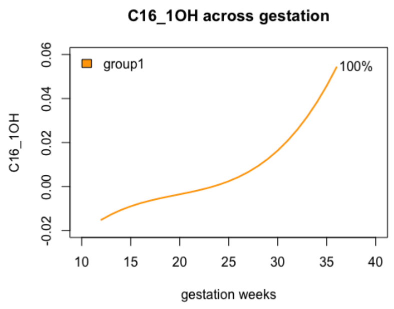
C16_OH	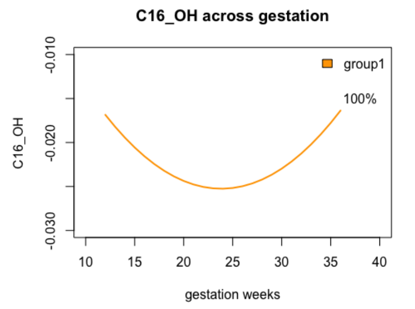	C18:2	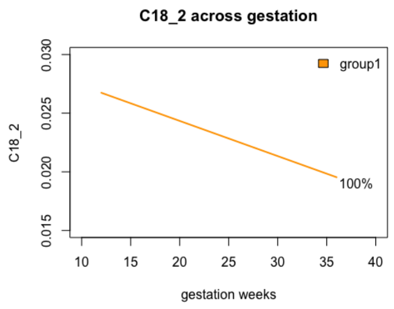

<table>
<tr>
<td>C18:1</td>
<td>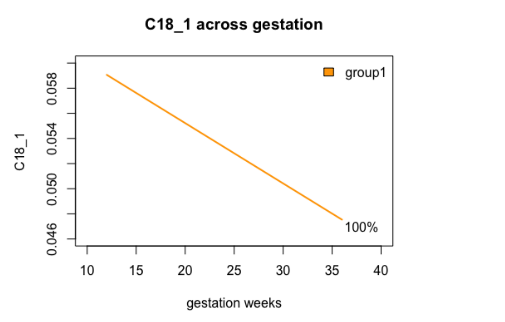
</td>
<td>C18</td>
<td>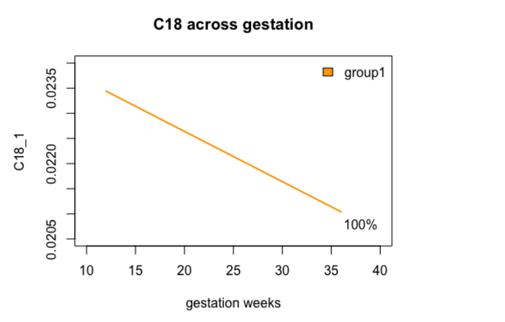
</td>
</tr>
</table>

[a] Trajectories were identified based on a weighted latent class model. We compared model fit for each biomarker based on 1–4 trajectory groups and fit linear, quadratic, and cubic models. Final models were selected based on Jeffreys's scale. Final selected models had ≥5% of the data in each identified group; [b] Group 1 shown in orange; Group 2 shown in blue; and Group 3 shown in green. Percentages represent the proportion of the data estimated to make up each group.

Supplementary Figure S2: Joint trajectory of acylcarnitine across gestational week

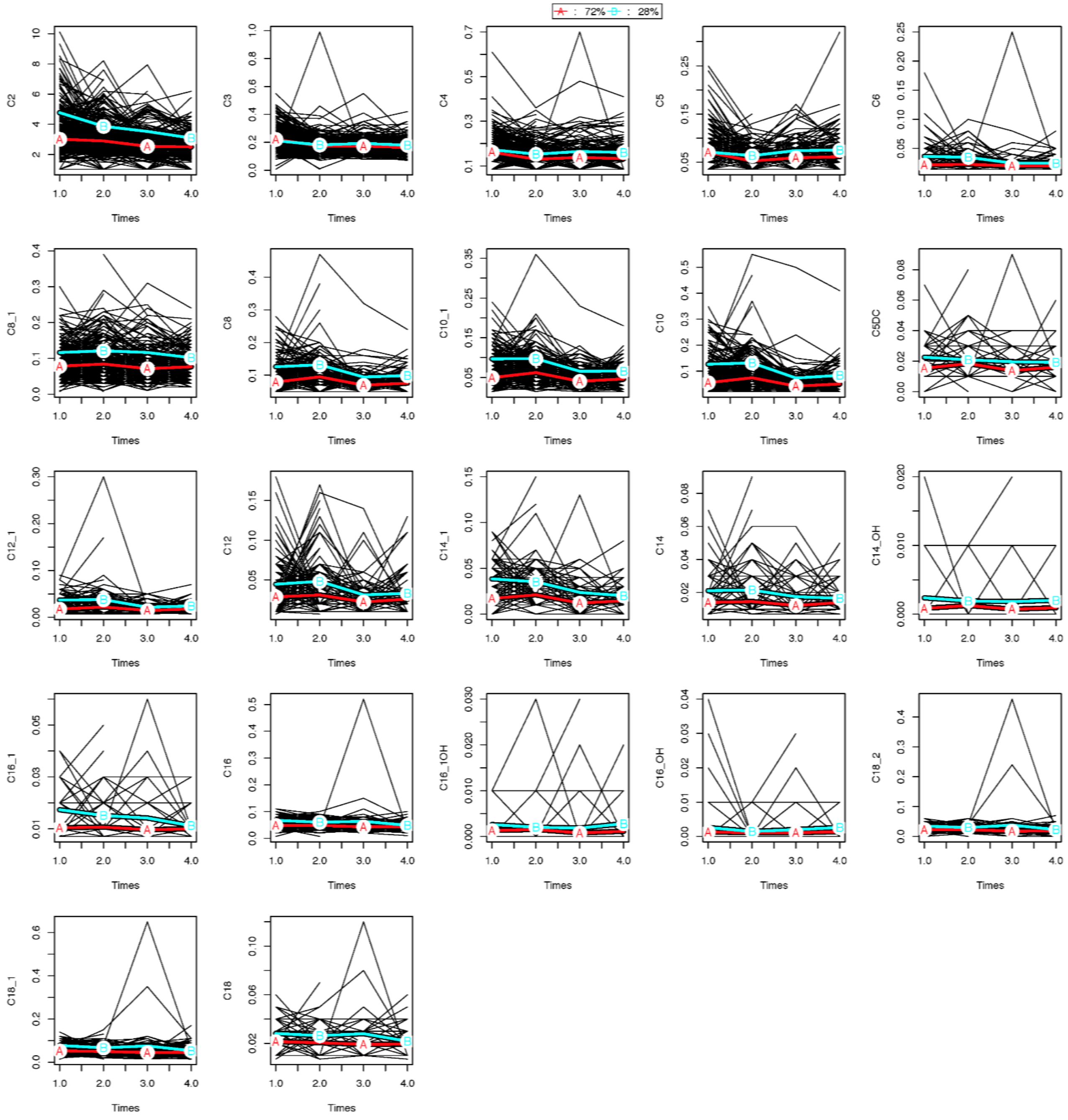

Supplementary Table S1. Acylcarnitine in association with neonatal birthweight, birthweight z score, length, sum of skinfolds and sum of body circumstance [a b c]

Maternal biomarker	Neonatal outcome, Adjusted β (95% CI)				
	Birthweight, g	Birthweight, z score	Length, cm	Sum of skinfolds, mm	Sum of circumference, cm
C2					
10–14 weeks	-0.07 (-0.32, 0.18)	0 (-0.14, 0.15)	0.01 (-0.19, 0.2)	-0.01 (-0.18, 0.17)	0.03 (-0.12, 0.18)
15–26 weeks	-0.19 (-0.42, 0.05)	-0.12 (-0.27, 0.03)	-0.07 (-0.29, 0.16)	0.04 (-0.14, 0.22)	-0.04 (-0.19, 0.11)
23–31 weeks	-0.04 (-0.19, 0.12)	-0.01 (-0.19, 0.17)	-0.06 (-0.2, 0.09)	0.01 (-0.19, 0.21)	0.02 (-0.16, 0.19)
33–39 weeks	-0.04 (-0.21, 0.13)	-0.04 (-0.23, 0.15)	-0.07 (-0.29, 0.14)	-0.03 (-0.22, 0.15)	0 (-0.18, 0.18)
C3					
10–14 weeks	0.08 (-0.07, 0.22)	0.07 (-0.06, 0.19)	0.09 (-0.05, 0.23)	0.11 (-0.04, 0.25)	0.09 (-0.05, 0.24)
15–26 weeks	-0.02 (-0.17, 0.13)	-0.03 (-0.15, 0.09)	0.04 (-0.1, 0.18)	0.21 (0.05, 0.37)	0.1 (-0.05, 0.25)
23–31 weeks	0 (-0.15, 0.15)	-0.01 (-0.17, 0.15)	-0.05 (-0.21, 0.11)	0.12 (0, 0.25)	0.04 (-0.12, 0.21)
33–39 weeks	-0.06 (-0.24, 0.13)	-0.1 (-0.28, 0.08)	-0.11 (-0.3, 0.09)	-0.07 (-0.26, 0.13)	0.03 (-0.18, 0.24)
C4					
10–14 weeks	0.07 (-0.08, 0.22)	0.01 (-0.13, 0.15)	0.03 (-0.12, 0.18)	0.12 (-0.06, 0.29)	0.06 (-0.1, 0.22)
15–26 weeks	0.03 (-0.14, 0.19)	-0.04 (-0.18, 0.1)	0.02 (-0.15, 0.18)	0.11 (-0.02, 0.25)	0.04 (-0.12, 0.19)
23–31 weeks	-0.09 (-0.27, 0.09)	-0.16 (-0.36, 0.03)	-0.31 (-0.56, -0.06)	-0.05 (-0.29, 0.19)	-0.07 (-0.25, 0.12)
33–39 weeks	-0.24 (-0.46, -0.02)	-0.26 (-0.46, -0.05)	-0.4 (-0.64, -0.16)	-0.24 (-0.45, -0.02)	-0.12 (-0.39, 0.14)
C5					
10–14 weeks	0.1 (-0.03, 0.24)	0.07 (-0.04, 0.19)	0.06 (-0.08, 0.2)	0.04 (-0.11, 0.19)	0.06 (-0.09, 0.22)
15–26 weeks	-0.02 (-0.19, 0.16)	0 (-0.13, 0.13)	-0.06 (-0.23, 0.11)	0.13 (0, 0.26)	-0.03 (-0.18, 0.13)
23–31 weeks	0.01 (-0.14, 0.15)	-0.04 (-0.2, 0.11)	-0.13 (-0.36, 0.09)	-0.04 (-0.21, 0.13)	0.08 (-0.08, 0.23)
33–39 weeks	-0.12 (-0.32, 0.08)	-0.11 (-0.35, 0.14)	-0.11 (-0.43, 0.21)	-0.02 (-0.29, 0.24)	-0.01 (-0.21, 0.18)
C6					
10–14 weeks	0.07 (-0.03, 0.18)	0.05 (-0.07, 0.16)	0.02 (-0.11, 0.15)	0.16 (0.02, 0.29)	0 (-0.12, 0.13)
15–26 weeks	-0.04 (-0.26, 0.17)	0.08 (-0.06, 0.22)	-0.06 (-0.24, 0.13)	0.14 (-0.01, 0.28)	0.1 (-0.03, 0.23)
23–31 weeks	-0.07 (-0.12, -0.02)	-0.1 (-0.16, -0.04)	-0.05 (-0.12, 0.01)	-0.04 (-0.12, 0.04)	-0.07 (-0.14, 0.01)
33–39 weeks	-0.03 (-0.13, 0.07)	-0.04 (-0.13, 0.05)	0 (-0.11, 0.1)	-0.14 (-0.26, -0.03)	-0.02 (-0.13, 0.09)
C8:1					
10–14 weeks	-0.05 (-0.26, 0.16)	0.03 (-0.11, 0.16)	0.04 (-0.16, 0.24)	-0.1 (-0.28, 0.09)	-0.08 (-0.27, 0.11)
15–26 weeks	-0.11 (-0.32, 0.11)	-0.05 (-0.21, 0.11)	0.08 (-0.13, 0.29)	0.04 (-0.18, 0.25)	-0.03 (-0.21, 0.15)
23–31 weeks	0.09 (-0.03, 0.2)	0.06 (-0.1, 0.21)	0.03 (-0.12, 0.17)	0.08 (-0.04, 0.21)	0.12 (0.01, 0.24)
33–39 weeks	0.05 (-0.16, 0.26)	0.04 (-0.16, 0.23)	-0.04 (-0.24, 0.16)	-0.06 (-0.23, 0.11)	0.03 (-0.19, 0.26)
C8					
10–14 weeks	0.02 (-0.12, 0.15)	0.02 (-0.09, 0.14)	0.01 (-0.14, 0.15)	0.12 (-0.03, 0.26)	-0.05 (-0.18, 0.09)
15–26 weeks	-0.07 (-0.23, 0.1)	0.01 (-0.1, 0.12)	-0.07 (-0.23, 0.1)	0.12 (-0.02, 0.25)	0.01 (-0.11, 0.13)
23–31 weeks	0.17 (-0.01, 0.35)	0.22 (0.04, 0.39)	0.19 (0.04, 0.34)	0.27 (0.12, 0.42)	0.21 (0.02, 0.39)
33–39 weeks	0.08 (-0.07, 0.24)	0.1 (-0.06, 0.25)	0.11 (-0.11, 0.33)	0.23 (0.05, 0.41)	-0.04 (-0.22, 0.14)
C10:1					
10–14 weeks	0.02 (-0.15, 0.2)	0.04 (-0.08, 0.16)	0.08 (-0.09, 0.25)	0.12 (-0.04, 0.28)	-0.01 (-0.14, 0.13)
15–26 weeks	-0.08 (-0.29, 0.12)	-0.03 (-0.16, 0.1)	-0.09 (-0.28, 0.09)	0.12 (-0.06, 0.3)	0.03 (-0.09, 0.15)
23–31 weeks	0.08 (-0.08, 0.25)	0.14 (-0.03, 0.32)	0.11 (-0.11, 0.32)	0.21 (0.05, 0.37)	0.12 (-0.06, 0.3)
33–39 weeks	0.13 (-0.02, 0.28)	0.1 (-0.07, 0.27)	0.07 (-0.12, 0.26)	0.12 (-0.07, 0.3)	0.02 (-0.14, 0.19)
C10					
10–14 weeks	0.05 (-0.1, 0.19)	0.03 (-0.09, 0.15)	0.05 (-0.11, 0.21)	0.15 (0, 0.29)	-0.02 (-0.16, 0.12)
15–26 weeks	-0.1 (-0.29, 0.09)	-0.04 (-0.16, 0.08)	-0.1 (-0.27, 0.07)	0.1 (-0.05, 0.25)	-0.02 (-0.14, 0.11)
23–31 weeks	0 (-0.12, 0.13)	0.07 (-0.08, 0.21)	0.1 (-0.06, 0.25)	0.14 (0.02, 0.26)	0.04 (-0.1, 0.19)

33–39 weeks	-0.03 (-0.18, 0.13)	-0.01 (-0.21, 0.19)	0.08 (-0.12, 0.28)	0.08 (-0.12, 0.28)	-0.07 (-0.31, 0.16)
C5:DC					
10–14 weeks	0.01 (-0.13, 0.14)	0.02 (-0.08, 0.12)	0.05 (-0.08, 0.19)	-0.01 (-0.16, 0.13)	-0.01 (-0.15, 0.14)
15–26 weeks	-0.12 (-0.36, 0.12)	-0.06 (-0.18, 0.06)	-0.06 (-0.2, 0.09)	-0.05 (-0.19, 0.09)	0.05 (-0.08, 0.18)
23–31 weeks	0.06 (-0.16, 0.28)	0.06 (-0.17, 0.29)	0.05 (-0.19, 0.3)	0.12 (-0.1, 0.34)	0.18 (-0.07, 0.42)
33–39 weeks	0.11 (-0.1, 0.32)	0.09 (-0.14, 0.32)	0.12 (-0.1, 0.34)	0.03 (-0.17, 0.24)	0.14 (-0.09, 0.36)
C12:1					
10–14 weeks	-0.02 (-0.2, 0.17)	0.02 (-0.11, 0.15)	0.01 (-0.15, 0.18)	0.13 (-0.06, 0.33)	0 (-0.14, 0.13)
15–26 weeks	-0.15 (-0.38, 0.08)	-0.07 (-0.17, 0.03)	-0.07 (-0.21, 0.06)	0.13 (-0.06, 0.31)	-0.07 (-0.13, 0)
23–31 weeks	0.08 (-0.11, 0.26)	0.17 (-0.01, 0.36)	0.12 (-0.06, 0.3)	0.16 (-0.02, 0.35)	0.11 (-0.08, 0.31)
33–39 weeks	0.08 (-0.07, 0.23)	0.04 (-0.11, 0.2)	0.09 (-0.11, 0.29)	0.07 (-0.1, 0.24)	0 (-0.16, 0.16)
C12					
10–14 weeks	0.02 (-0.15, 0.18)	0.03 (-0.08, 0.14)	0.02 (-0.12, 0.16)	0.12 (-0.03, 0.26)	0.01 (-0.14, 0.15)
15–26 weeks	-0.23 (-0.46, -0.01)	-0.1 (-0.22, 0.01)	-0.15 (-0.3, 0)	-0.01 (-0.15, 0.13)	-0.05 (-0.21, 0.11)
23–31 weeks	0.18 (-0.01, 0.37)	0.21 (-0.02, 0.44)	0.07 (-0.13, 0.28)	-0.02 (-0.34, 0.3)	0.27 (0.11, 0.44)
33–39 weeks	0.04 (-0.12, 0.21)	0.05 (-0.11, 0.2)	0.11 (-0.1, 0.33)	0.1 (-0.1, 0.3)	-0.11 (-0.3, 0.07)
C14:1					
10–14 weeks	0.01 (-0.21, 0.22)	0.07 (-0.07, 0.2)	0.01 (-0.19, 0.21)	0.11 (-0.11, 0.32)	0.06 (-0.08, 0.2)
15–26 weeks	-0.25 (-0.38, -0.12)	-0.1 (-0.19, -0.02)	-0.12 (-0.22, -0.02)	0.05 (-0.15, 0.24)	-0.07 (-0.21, 0.08)
23–31 weeks	0.04 (-0.07, 0.14)	0.05 (-0.08, 0.18)	-0.05 (-0.16, 0.07)	-0.16 (-0.32, 0)	0.11 (0.02, 0.21)
33–39 weeks	0.15 (0.01, 0.29)	0.14 (-0.01, 0.29)	0.16 (-0.02, 0.35)	0.06 (-0.13, 0.25)	0.15 (0, 0.31)
C14					
10–14 weeks	0.04 (-0.09, 0.16)	0.03 (-0.08, 0.15)	0.02 (-0.13, 0.18)	0.07 (-0.08, 0.23)	0 (-0.13, 0.14)
15–26 weeks	-0.24 (-0.45, -0.04)	-0.11 (-0.22, 0.01)	-0.13 (-0.26, 0)	-0.06 (-0.21, 0.08)	-0.07 (-0.22, 0.08)
23–31 weeks	0.18 (-0.01, 0.38)	0.2 (-0.02, 0.41)	0.07 (-0.14, 0.27)	-0.06 (-0.36, 0.24)	0.25 (0.08, 0.42)
33–39 weeks	0.03 (-0.18, 0.24)	0.05 (-0.15, 0.26)	0.08 (-0.21, 0.37)	0.2 (-0.01, 0.41)	-0.06 (-0.26, 0.15)
C14 _OH					
10–14 weeks	0.08 (-0.04, 0.2)	0.05 (-0.08, 0.18)	0.12 (0.01, 0.23)	0.05 (-0.08, 0.18)	0.04 (-0.12, 0.19)
15–26 weeks	-0.04 (-0.27, 0.19)	0.05 (-0.1, 0.19)	0.02 (-0.17, 0.21)	0.02 (-0.16, 0.21)	0.05 (-0.11, 0.21)
23–31 weeks	0.07 (-0.05, 0.19)	0 (-0.16, 0.15)	0.03 (-0.11, 0.16)	-0.09 (-0.31, 0.13)	0.08 (-0.08, 0.23)
33–39 weeks	0.18 (-0.02, 0.38)	0.19 (0, 0.38)	0.18 (0.01, 0.36)	0.09 (-0.08, 0.25)	0.18 (0, 0.36)
C16_1					
10–14 weeks	0.01 (-0.19, 0.21)	0.04 (-0.1, 0.18)	-0.02 (-0.22, 0.17)	0.13 (-0.06, 0.31)	-0.01 (-0.14, 0.13)
15–26 weeks	-0.17 (-0.41, 0.06)	-0.05 (-0.18, 0.09)	-0.07 (-0.26, 0.11)	0.09 (-0.09, 0.28)	0.03 (-0.09, 0.14)
23–31 weeks	0.02 (-0.08, 0.12)	0.02 (-0.09, 0.13)	0.04 (-0.13, 0.2)	-0.13 (-0.32, 0.06)	0.09 (-0.01, 0.2)
33–39 weeks	0.07 (-0.08, 0.22)	0.04 (-0.14, 0.22)	0.1 (-0.1, 0.3)	0.04 (-0.18, 0.25)	0.04 (-0.14, 0.23)
C16					
10–14 weeks	-0.06 (-0.24, 0.13)	0.02 (-0.1, 0.14)	0.01 (-0.19, 0.21)	0.05 (-0.12, 0.23)	-0.03 (-0.16, 0.09)
15–26 weeks	-0.13 (-0.32, 0.06)	-0.07 (-0.19, 0.06)	-0.12 (-0.29, 0.04)	0.03 (-0.13, 0.2)	-0.04 (-0.16, 0.08)
23–31 weeks	-0.02 (-0.09, 0.04)	-0.03 (-0.09, 0.03)	-0.06 (-0.13, 0)	-0.18 (-0.26, -0.09)	0.05 (-0.01, 0.11)
33–39 weeks	-0.19 (-0.35, -0.02)	-0.11 (-0.28, 0.06)	-0.11 (-0.32, 0.1)	-0.07 (-0.23, 0.1)	-0.17 (-0.35, 0)
C16_1OH					
10–14 weeks	-0.1 (-0.32, 0.11)	-0.02 (-0.17, 0.13)	-0.11 (-0.28, 0.07)	-0.03 (-0.18, 0.11)	0.11 (-0.04, 0.26)
15–26 weeks	-0.05 (-0.3, 0.2)	0.06 (-0.08, 0.21)	-0.02 (-0.21, 0.18)	0.07 (-0.08, 0.23)	0.07 (-0.04, 0.19)
23–31 weeks	-0.04 (-0.22, 0.14)	-0.07 (-0.27, 0.13)	-0.18 (-0.33, -0.03)	-0.25 (-0.46, -0.03)	-0.04 (-0.28, 0.2)
33–39 weeks	0.11 (-0.06, 0.28)	0.06 (-0.11, 0.23)	0.17 (0.02, 0.32)	0.16 (-0.09, 0.41)	0.02 (-0.19, 0.22)
C16_OH					
10–14 weeks	-0.04 (-0.25, 0.17)	0.07 (-0.06, 0.19)	-0.01 (-0.15, 0.12)	0.07 (-0.06, 0.2)	0.05 (-0.1, 0.2)

15–26 weeks	-0.17 (-0.42, 0.08)	-0.03 (-0.18, 0.13)	-0.15 (-0.31, 0.02)	-0.08 (-0.16, 0.01)	0.03 (-0.1, 0.15)
23–31 weeks	0.11 (-0.06, 0.28)	0.13 (-0.04, 0.3)	0 (-0.17, 0.17)	-0.01 (-0.13, 0.11)	0.12 (-0.13, 0.38)
33–39 weeks	0.27 (0.1, 0.44)	0.26 (0.08, 0.43)	0.22 (0.08, 0.37)	0.3 (0.11, 0.49)	0.22 (0.03, 0.42)
C18:2					
10–14 weeks	-0.04 (-0.19, 0.1)	0.02 (-0.1, 0.15)	-0.01 (-0.2, 0.18)	0.06 (-0.11, 0.22)	-0.02 (-0.16, 0.12)
15–26 weeks	-0.13 (-0.33, 0.07)	-0.08 (-0.21, 0.05)	-0.11 (-0.27, 0.05)	0.04 (-0.13, 0.21)	0.02 (-0.11, 0.14)
23–31 weeks	-0.02 (-0.08, 0.04)	-0.02 (-0.08, 0.03)	-0.09 (-0.16, -0.01)	-0.21 (-0.28, -0.13)	0.07 (0.02, 0.12)
33–39 weeks	-0.02 (-0.19, 0.16)	0.02 (-0.17, 0.2)	0.1 (-0.1, 0.29)	-0.01 (-0.2, 0.17)	-0.02 (-0.22, 0.18)
C18:1					
10–14 weeks	0.05 (-0.12, 0.21)	0.08 (-0.06, 0.21)	0.11 (-0.09, 0.3)	0.06 (-0.1, 0.22)	0.01 (-0.12, 0.15)
15–26 weeks	-0.08 (-0.28, 0.13)	0.02 (-0.12, 0.15)	-0.03 (-0.2, 0.15)	-0.02 (-0.2, 0.16)	0.01 (-0.12, 0.14)
23–31 weeks	-0.01 (-0.07, 0.05)	-0.02 (-0.08, 0.05)	-0.05 (-0.12, 0.03)	-0.19 (-0.28, -0.11)	0.07 (0, 0.13)
33–39 weeks	0.04 (-0.14, 0.21)	0.04 (-0.12, 0.2)	0.19 (0.02, 0.36)	0.01 (-0.17, 0.18)	0.04 (-0.14, 0.23)
C18					
10–14 weeks	-0.01 (-0.14, 0.11)	-0.03 (-0.15, 0.09)	0 (-0.14, 0.14)	0.01 (-0.12, 0.13)	-0.08 (-0.22, 0.07)
15–26 weeks	0 (-0.15, 0.16)	0.08 (-0.05, 0.22)	-0.01 (-0.16, 0.14)	0.07 (-0.07, 0.22)	0.12 (-0.02, 0.27)
23–31 weeks	0 (-0.1, 0.1)	0.02 (-0.09, 0.12)	-0.02 (-0.14, 0.1)	-0.12 (-0.32, 0.07)	0.1 (-0.01, 0.21)
33–39 weeks	0.11 (-0.07, 0.3)	0.14 (-0.03, 0.3)	0.13 (-0.07, 0.34)	0.13 (-0.04, 0.31)	0.06 (-0.15, 0.27)

[a] Adjusted for maternal age (continuous), race/ethnicity (non-Hispanic white, non-Hispanic black, Hispanic, Asian), education (high school or less, some college/associate degree, 4-year college degree or higher), nulliparity (yes/no), prepregnancy body mass index (continuous), gestational age at blood collection (continuous); [b] The coefficient and 95% CI are standardized estimates, representing outcome change per one standard deviation increase in acylcarnitines

Supplementary Table S2: Joint trajectory of acylcarnitines across gestation and the association with neonatal birthweight, birthweight z score, length, sum of skinfolds, and sum of body circumstances

**Maternal biomarker**	**Neonatal outcome, Adjusted β (95% CI)**				
	**Birthweight, g**	**Birthweight, z-score**	**Length, cm**	**Sum of skinfolds, mm**	**Sum of circumference, cm**
Trajectory					
Group 2	1340 (-1.64, 269)	0.19 (-0.06, 0.44)	0.52 (-0.55, 1.58)	0.72 (-1.48, 2.91)	1.82 (-0.92, 4.57)
Group 1	0.0 (ref)	0.0 (ref)	0.0 (ref)	0.0 (ref)	0.0 (ref)
